# Concerted Efforts to Control or Eliminate Neglected Tropical Diseases: How Much Health Will Be Gained?

**DOI:** 10.1371/journal.pntd.0004386

**Published:** 2016-02-18

**Authors:** Sake J. de Vlas, Wilma A. Stolk, Epke A. le Rutte, Jan A. C. Hontelez, Roel Bakker, David J. Blok, Rui Cai, Tanja A. J. Houweling, Margarete C. Kulik, Edeltraud J. Lenk, Marianne Luyendijk, Suzette M. Matthijsse, William K. Redekop, Inge Wagenaar, Julie Jacobson, Nico J. D. Nagelkerke, Jan H. Richardus

**Affiliations:** 1 Department of Public Health, Erasmus MC, University Medical Center Rotterdam, Rotterdam, The Netherlands; 2 Center for Tobacco Control Research and Education, University of California at San Francisco, San Francisco, California, United States of America; 3 Institute of Health Policy and Management, Erasmus University Rotterdam, Rotterdam, The Netherlands; 4 Bill and Melinda Gates Foundation, Seattle, Washington, United States of America; University of Florida, UNITED STATES

## Abstract

**Background:**

The London Declaration (2012) was formulated to support and focus the control and elimination of ten neglected tropical diseases (NTDs), with targets for 2020 as formulated by the WHO Roadmap. Five NTDs (lymphatic filariasis, onchocerciasis, schistosomiasis, soil-transmitted helminths and trachoma) are to be controlled by preventive chemotherapy (PCT), and four (Chagas’ disease, human African trypanosomiasis, leprosy and visceral leishmaniasis) by innovative and intensified disease management (IDM). Guinea worm, virtually eradicated, is not considered here. We aim to estimate the global health impact of meeting these targets in terms of averted morbidity, mortality, and disability adjusted life years (DALYs).

**Methods:**

The Global Burden of Disease (GBD) 2010 study provides prevalence and burden estimates for all nine NTDs in 1990 and 2010, by country, age and sex, which were taken as the basis for our calculations. Estimates for other years were obtained by interpolating between 1990 (or the start-year of large-scale control efforts) and 2010, and further extrapolating until 2030, such that the 2020 targets were met. The NTD disease manifestations considered in the GBD study were analyzed as either reversible or irreversible. Health impacts were assessed by comparing the results of achieving the targets with the counterfactual, construed as the health burden had the 1990 (or 2010 if higher) situation continued unabated.

**Principle Findings/Conclusions:**

Our calculations show that meeting the targets will lead to about 600 million averted DALYs in the period 2011–2030, nearly equally distributed between PCT and IDM-NTDs, with the health gain amongst PCT-NTDs mostly (96%) due to averted disability and amongst IDM-NTDs largely (95%) from averted mortality. These health gains include about 150 million averted irreversible disease manifestations (e.g. blindness) and 5 million averted deaths. Control of soil-transmitted helminths accounts for one third of all averted DALYs. We conclude that the projected health impact of the London Declaration justifies the required efforts.

## Author Summary

Neglected tropical diseases (NTDs) are a group of infectious diseases that occur mostly in poor, warm countries. NTDs are caused by various bacteria and parasites, such as worms. They can either be cured or prevented through drugs and other interventions, such as control of insects that spread the infection. The London Declaration is a statement by various organizations, including the World Health Organization (WHO) and pharmaceutical companies that donate the necessary drugs. The declaration endorses targets for disease reductions by 2020, as recently formulated in the WHO Roadmap, to be achieved by rigorous application of available interventions. We explore how much health can be gained if these targets are indeed achieved. We estimate that in such case 5 million deaths can be averted before 2030 and also that huge reductions in ill-health and disability can be realized. Over the period 2011–2030, a total health gain would be accomplished of about 600 million disability adjusted life years (DALYs) averted. DALYs are a measure of disease burden, consisting of life years lost and years lived with disability. This enormous health gain seems to justify similar investments as for e.g. HIV or malaria control.

## Introduction

Neglected tropical diseases (NTDs) are considered a special category of infectious diseases, distinct from the major killers HIV, tuberculosis and malaria, which have been the main focus of attention and funding for developing countries over the past decades. NTDs are largely confined to (sub)tropical resource-constrained regions, where they cause substantial morbidity, disability and even mortality, as documented by the recent Global Burden of Disease (GBD) estimates [1–4], and consequently have high socioeconomic impact [5,6]. Most NTDs are either curable or preventable, but in practice there exist major barriers to the effective implementation of control. Fortunately, international commitment to NTD control has rapidly increased in recent years. In 2012, the World Health Organization (WHO) formulated a ‘Roadmap’ towards ambitious control and elimination targets [7]. By endorsing the London Declaration on NTDs, several private and public sector organizations committed to meet those targets [8]. For five NTDs—lymphatic filariasis (LF), onchocerciasis, schistosomiasis, soil-transmitted helminths (STH) and trachoma—the primary control strategy is preventive chemotherapy (PCT). For four other NTDs—Chagas’ disease, human African trypanosomiasis (HAT), leprosy and visceral leishmaniasis (VL)–control programs rely on case detection with innovative and intensified disease management (IDM), sometimes in combination with other measures such as vector control. Guinea worm (dracunculiasis) is confined to just a few residual foci in Africa and close to being eradicated. For LF, trachoma, HAT and leprosy the target is elimination by 2020, and for the others it is currently control [7,9].

The London Declaration was formulated to accelerate progress towards the WHO Roadmap targets by sustaining or expanding existing drug donation initiatives; providing funding to support NTD programs, strengthen drug distribution, and research and development; and enhancing collaboration and coordination on NTDs at (inter)national levels [8]. To further motivate and justify these efforts it is important to know their expected health gains. We therefore aim to estimate the global health impact of meeting the WHO Roadmap targets in terms of averted morbidity and mortality, expressed in years lived with disability (YLD), years of life lost (YLL), and disability adjusted life years (DALYs). YLD reflects the number of prevalent cases of each considered disease manifestation multiplied by a disease-specific disability weight between 0 (perfect health) and 1 (equivalent to death), whereas YLL reflects the number of deaths times a standard life expectancy at the age of death in years. The number of DALYs is the sum of both measures (DALYs = YLD + YLL).

## Methods

### Data sources

Two datasets were used in our calculations. First, the GBD-2010 estimates regarding NTDs were made available to us by the Institute for Health Metrics and Evaluation (IHME), Seattle, USA [3,10]. Second, UNPOP demographic data and projections were obtained from the website of United Nations Department of Economic and Social affairs [11]. The GBD-2010 data consist of three burden estimates: prevalent cases, years lived with disability (YLD) and years of life lost (YLL). These estimates were available for 1990 and 2010, per country, age group and sex. Prevalent cases were provided per disease manifestation (sequela), whereas YLD and YLL were only provided as totals per NTD. Table 1 gives an overview of all 31 sequelae considered in the GBD calculations for the London Declaration NTDs. Guinea worm was not included in the GBD study and is therefore not considered here. For STH, burden estimates were available for ascariasis, hookworm disease and trichuriasis separately. Background documents justifying and describing the underlying assumptions of the GBD estimates, including disability weights, were also kindly made available to us. GBD estimates were structured according to the following age groups: 0–6 days, 7–27 days, 28–364 days, 1–4 years, 5–9 years, …, 75–79 years, and 80+ years. We combined the four youngest age groups into a 0–4 years group. For irreversible sequelae (see below), the number of prevalent cases was redistributed into 1-year age groups, using a smoothing method that minimizes the squared differences between successive years, under the constraint that 5-years totals equal the available data. The demographic data were already available in 1-year age groups.

**Table 1:** The 31 sequelae (categorized as either reversible or irreversible) and associated mortality in the Global Burden of Disease 2010 study for the ten London Declaration NTDs, except Guinea worm. The bold numbers reflect the years lived with disability (YLD) and years of life lost (YLL) for each NTD in 2010, as estimated by the GBD 2010 study [1–4]. The excess mortality rate (μ*) was chosen to reflect the severity of the sequela. The average disability weights were used to relate YLD to prevalent cases in our calculations for NTDs with multiple sequelae. Salomon et al. [24] provide more information about disability weights and lay explanations of sequelae. (a) The original GBD value for LF was 2.74 million YLD, but as Cambodia, Federated States of Micronesia, Maldives, Samoa, Sri Lanka, Togo, Tonga, Vanuatu, and Vietnam had reached elimination before 2010, their remaining burden (total of 0.04 million YLD) was removed from our calculations. (b) The GBD values for leprosy were based on a recalculation; see methods section. (c) A disability weight (DW = 0.097) for visceral leishmaniasis was needed to distinguish it from cutaneous leishmaniasis (DW = 0.013), as both were combined as leishmaniasis in the YLD values available from GBD; YLL due to leishmaniasis was assumed to be fully caused by visceral leishmaniasis.

NTD (YLD and YLL in millions from the Global Burden of Disease 2010 study)			
Sequela	Reversible/Irreversible	Excess mortality rate (μ*)	Average disability weight
**Lymphatic filariasis (YLD: 2.70)** ^**a**^			
Lymphedema	Irreversible	0.0	0.110
Hydrocele due to lymphatic filariasis	Irreversible	0.0	0.097
**Onchocerciasis (YLD: 0.49)**			
Skin disease due to onchocerciasis	Reversible	NA	0.079
Vision loss due to onchocerciasis	Irreversible	0.05	0.101
**Schistosomiasis (YLD: 2.99, YLL: 0.32)**			
Schistosomiasis (i.e. symptomatic infection)	Reversible	NA	0.005
Mild diarrhea due to schistosomiasis	Reversible	NA	0.061
Anemia due to schistosomiasis	Reversible	NA	0.036
Hepatomegaly due to schistosomiasis	Reversible	NA	0.012
Hematemesis due to schistosomiasis	Irreversible	0.05	0.323
Ascites due to schistosomiasis	Irreversible	0.05	0.123
Dysuria due to schistosomiasis	Reversible	NA	0.012
Bladder pathology due to schistosomiasis	Irreversible	0.05	0.012
Hydronephrosis due to schistosomiasis	Reversible	NA	0.012
**STH—Ascariasis (YLD: 1.11, YLL: 0.20)**			
Ascariasis infestation	Reversible	NA	0.030
Severe wasting due to ascariasis	Reversible	NA	0.127
Mild abdominopelvic problems due to ascariasis	Reversible	NA	0.012
**STH—Hookworm disease (YLD: 3.19)**			
Hookworm infestation	Reversible	NA	0.030
Severe wasting due to hookworm disease	Reversible	NA	0.127
Mild abdominopelvic problems due to hookworm disease	Reversible	NA	0.012
Anemia due to hookworm disease	Reversible	NA	0.032
**STH—Trichuriasis (YLD: 0.64)**			
Trichuriasis infestation	Reversible	NA	0.030
Severe wasting due to trichuriasis	Reversible	NA	0.127
Mild abdominopelvic problems due to trichuriasis	Reversible	NA	0.012
**Trachoma (YLD: 0.33)**			
Trachoma	Irreversible	0.05	-
**Chagas’ disease (YLD: 0.31, YLL: 0.24)**			
Acute Chagas’ disease	Reversible	NA	0.053
Chronic heart disease due to Chagas’ disease	Irreversible	0.10	0.078
Chronic digestive disease due to Chagas’ disease	Irreversible	0.0	0.078
Heart failure due to Chagas’ disease	Irreversible	0.10	0.139
**Human African trypanosomiasis (YLD: 0.01, YLL: 0.55)**			
African trypanosomiasis	Reversible	NA	-
**Leprosy (YLD: 0.04)** ^**b**^			
Disfigurement due to leprosy	Irreversible	0.0	-
**Visceral leishmaniasis (YLD: 0.01, YLL: 3.19)**			
Visceral leishmaniasis	Reversible	NA	0.097 ^c^

### General approach

The GBD estimates of the number of prevalent cases for all 31 sequelae and 5 causes of death (HAT, VL, STH-ascariasis, Chagas’ disease and schistosomiasis) in 1990 and 2010 were taken as the basis for our calculations. Estimates for other years were obtained by interpolating between 1990 and 2010, and further extrapolated until 2030, under the assumption that the 2020 WHO Roadmap targets were met and sustained beyond 2020. Health impacts were assessed by comparing the results of achieving the targets with the counterfactual, construed as the health burden had the 1990 situation continued unabated. Prevalent cases (both remaining and counterfactual) were translated to YLD and YLL, and summed to arrive at DALYs. The health impact of reaching the targets was expressed as DALYs averted over the decades 2011–2020 and 2021–2030.

All calculations were carried out in duplicate in Microsoft Excel, and verified using R. All results (totals and country-specific values), underlying calculations and assumptions are available as an open-access web-based dissemination tool (https://erasmusmcmgz.shinyapps.io/dissemination/). A detailed step-wise explanation of our methodology is given below.

### Trends for reversible and irreversible sequelae

Sequelae were first categorized as either reversible or irreversible (Table 1), depending on whether treatment of the underlying infection would remove the sequelae in a relatively short time, say, within a couple of years at most. For all reversible sequelae, interventions were considered to affect their prevalence, while for irreversible sequelae this was their incidence. Linear interpolation (at the log-scale for irreversible sequelae) was carried out between 1990 (or the start-year of large-scale control efforts) and 2010 for prevalence rates (i.e. the number of prevalent cases divided by population size) per sequela, country, age group and sex. Absolute numbers were then calculated from these interpolated prevalence rates, using the demographic UNPOP data. For 2020 (and beyond), WHO Roadmap targets were interpreted in terms of prevalence (for reversible sequelae) or incidence (for irreversible sequelae) levels, based on discussions with—mostly WHO—disease experts (Table 2). Trends in incidence and prevalence during the intervening years (usually 2010–2020) were obtained through linear interpolation between the 2010 levels (GBD data) and the interpreted targets. We then translated the calculated trends into absolute numbers of remaining cases using UNPOP projections for the period 2011–2030, and compared this with the counterfactual situation of no additional control efforts, to assess the impact of meeting the targets. The counterfactual was construed as the health burden that would have been expected had the 1990 epidemiological situation (i.e. disease incidence or prevalence) continued unabated. Whenever the 2010 prevalence of a sequela exceeded that of 1990, we took 2010 as the counterfactual.

**Table 2:** Interpretations of WHO Roadmap targets [7] as used in our calculations. All country-specific assumptions for each NTD are provided here: https://erasmusmcmgz.shinyapps.io/dissemination/.

Lymphatic filariasis	**Target:** By 2020, 70% of countries will have been verified as free of transmission and 30% will have entered post-intervention surveillance.
	**Interpretation:** The PCT database [25] provides start and end years of the intervention program per country. The incidence of both chronic manifestations (lymphedema and hydrocele) is assumed to linearly decrease to zero, one year before the anticipated last treatment round in each country.
Onchocerciasis	**Target:** To eliminate onchocerciasis where feasible (without a specified target year).
	**Interpretation:** Interventions will continue until the end year of interventions as estimated by APOC [26]; prevalence of skin disease and incidence of vision loss reach zero two years before the end year of interventions.
Schistosomiasis	**Target:** Elimination of transmission in certain regions and countries by 2015 or 2020. Global elimination in 2025 as a public health problem. In 2020, 75% national coverage is reached in all the countries requiring preventive chemotherapy for schistosomiasis.
	**Interpretation:** Global elimination in 2025, therefore in all countries prevalence of reversible and incidence of irreversible sequelae will go down to zero in 2025. The general start year of interventions is 2001, the same year as the WHA resolution on STH and schistosomiasis [27].
STH (ascariasis, hookworm disease and trichuriasis)	**Target:** 100% of countries requiring preventive chemotherapy for STH have achieved 75% national mass drug administration coverage of school-aged children (SAC) and pre-SAC by 2020.
	**Interpretation:** The pre-SAC and SAC (ages 5–14) will have 0% prevalence of morbidity by 2025. There will be 10% remaining prevalence of morbidity in the non-treated groups (0–4 and 15+) by the year 2025, relative to the STH level in 2010. Mortality due to ascariasis will be 0% in 2025, for all age groups. The general starting year of interventions is 2001, the same year as the WHA resolution on STH and schistosomiasis [27].
Trachoma	**Target:** Global elimination as a public health problem in 2020. All countries will have achieved the ultimate intervention goal and be free from blinding trachoma as a public health problem.
	**Interpretation:** Incidence of vision loss caused by trachoma will go down to zero in country specific years. Three WHO documents [7,28,29] provide most start years of intervention and target years of elimination.
Chagas’ disease	**Target:** To eliminate transmission through blood transfusion in the America’s, Europe and Western Pacific by 2015. To eliminate peri-domiciliary infestation in Latin America by 2020, but surveillance and control of oral transmission and congenital infection need to be sustained.
	**Interpretation:** For acute Chagas’ disease the prevalence will linearly decrease to 10% of the GBD 2010 value in 2020, and remain 10% onwards. This 10% reflects remaining burden due to infections from the sylvatic cycle. For chronic heart disease, chronic digestive disease and heart failure, incidence in 2020 will be 10% of that in 2010, and remain 10% onwards. The GBD data about Chagas’ disease only concern countries in Latin America, so no specific assumptions are needed for the rest of the world.
	**Note:** There are great concerns about the reliability of the GBD figures, as well as the feasibility of the London Declaration and associated WHO targets [30].
HAT	**Target:** Achieve elimination of >90% of foci by 2020. The global number of new cases reported annually for 2020 is <2000.
	**Interpretation:** There are exactly 2000 cases in 2020, and this number will subsequently decline to 0 in 2030. From 2020 onwards, all remaining cases will be detected and treated, meaning that HAT mortality is zero in 2020 and beyond. The overall number of prevalent cases in 2020 is 2.5% of the level in 2010.
	**Explanation:** In 2010, 9103 people died because of HAT according to YLL data of GBD 2010. By multiplying the number of people that died in 2010 by 3 (the burden before dying is assumed to last on average for three years in the GBD calculations) we arrive at 27,307 prevalent cases in 2010 that will eventually die because of HAT. The total point prevalence of HAT in 2010 is provided by the GBD: 36,863. This means that about 75% (27,307 out of 36,863) of prevalent cases will eventually die, whereas the remaining 25% (9,554 out of 36,863) will survive. The 9,554 surviving prevalence cases multiplied by 2 results in 19,108 new detected and successfully treated cases in 2010 (given the GBD assumption that disease lasts 6 months before treatment). Thus, on a global level there will be a decrease from 19,108 new surviving cases in 2010 down to 2000 new surviving cases in 2020, so roughly a decrease to 10% of the level in 2010. This decrease applies to the number of surviving prevalent cases, which is 25% of the total. This means that the overall number of prevalent cases will go down to 10% times 25% = 2.5% of the level in 2010, as the 75% of cases that eventually die will become 0.
	**Note:** The number of new detected and treated cases (19,108) and the number of new cases that will die (9,554) adds up to 28,211 new cases in 2010, which is substantially higher than what is known in WHO records [31]: 7139 new reported cases in 2010.
Leprosy	**Target:** Global interruption of transmission by 2020. Reduction of grade 2 disabilities in newly detected cases to below 1/million population at global level by 2020.
	**Interpretation:** The incidence of disfigurement due to leprosy has decreased in 2020 to 37% of the level in 2010, and will further reduce to 0% in 2030, in order to account for the target of global interruption of transmission by 2020.
	**Explanation:** According to the 2010 GBD data, the incidence of all newly detected cases was 318,876, of which 6% (19,132) had grade 2 disability [12]. This is 2.7/1 million globally. According to the WHO target, this should be reduced to 1/1 million in 2020, representing a reduction to approximately 37% of the level in 2010, or 7,086 incident cases in 2020.
	**Note:** See main text for our recalculations to arrive at grade 2 disability prevalences.
Visceral leishmaniasis	**Target:** On the Indian subcontinent (ISC), 1/10,000 new cases at (sub)district level per year by 2020; globally, 100% detection and treatment of VL.
	**Interpretation:** On ISC there will be a prevalence reduction to 5% of the 2010 situation, which will remain at 5% until 2030. Elsewhere, the prevalence of 2010 will remain unaltered. Morbidity in 2020 will have become 25% (Africa), 0.3% (ISC) and 10% (elsewhere) of the level in 2010, and remain constant thereafter.
	**Explanation about prevalence on ISC**: WHO reports approximately 20/10,000 new VL cases per year on ISC in 2010. Therefore, the target of 1/10,000 will be a reduction to 5% of the 2010 situation. This 5% will also apply to the prevalent cases.
	**Explanation about trends in death:** In 2010, 51,485 people died because of VL according to YLL data of GBD 2010. Also, worldwide there were 67,721 prevalent cases, which correspond to 270,884 new cases, given the GBD-assumed 3 month average duration of VL. Thus, in 2010 on average 19% of the people with VL died globally. According to the WHO targets, death due to VL will decrease substantially, but it will not go down to zero, as current treatment is not 100% effective [32]. We assume that in Africa 5% of the people (even though detected and treated) with VL will die in 2020, 1% of the people with VL on the Indian subcontinent, and 2% elsewhere. This means that in Africa the relative number of deaths (and also YLL) will decrease to 5/19 = about 25% of the level in 2010. On the Indian subcontinent this will be 0.05 times 1/19 = about 0.3% of the level in 2010. Elsewhere, this will be 2/19 = about 10% of the level in 2010. The regional differences in mortality rates were based on discussions with the disease experts and particularly reflect differences in treatment efficacy and HIV-coinfection.

### Incidence and prevalence calculations for irreversible sequelae

Interpolation for irreversible sequelae, such as blindness as a result of onchocerciasis, was carried out at the level of incidence, because even after elimination of infection these sequelae will persist until the death of the last patient. The annual incidence density *λ*(*a*,*t*) at age *a* and calendar time *t* of an irreversible condition is given by the following equation

$$\frac{\partial s\left(a,t\right)}{\partial a}+\frac{\partial s\left(a,t\right)}{\partial t}=-\lambda \left(a,t\right)\cdot s\left(a,t\right)+\left[1-s\left(a,t\right)\right]\cdot \mu \left(a,t\right)\cdot s\left(a,t\right)$$

where *s*(*a*,*t*) denotes the susceptible fraction (i.e. 1 –prevalence) of the population and *μ*(*a*,*t*) the excess mortality rate among those affected. In a stable endemic situation (i.e. without cohort effect, thus *∂s*(*a*,*t*)/*∂t* = 0) and without excess mortality (i.e. *μ*(*a*,*t*) = 0), *λ*(*a*,*t*) can be obtained from a single cross-sectional survey by taking the differences in the logarithmic age profile of the fraction susceptible. However, because the cross-sectional age profiles of GBD 1990 and 2010 for each sequela differed, we annualized the differences (on a logarithmic scale) in these profiles to obtain an estimate for *∂s*(*a*,*t*)/*∂t*. We further assumed the excess mortality rate to be independent of age and calendar time, and have a pre-set value μ* = 0.0, 0.05 or 0.10, dependent on the severity of the sequela (Table 1). The value of μ* was chosen after consultation of the disease experts and crudely reflected the mortality rates as used in the GBD calculations. The resulting incidences were calculated back to prevalences (of remaining cases) by ‘exposing’ cohorts to the derived age and time-specific incidence densities and excess mortality rates.

### Morbidity calculations: Years living with disability (YLD)

Predicted prevalent cases for each sequela were then translated to YLD, using two matrices of multiplication factors (one for the year 1990 and one for 2010) that we had derived from the GBD data as follows. Whenever an NTD had one sequela (e.g. trachoma), the GBD YLDs in 1990 and 2010 were divided by the number of prevalent cases in the same year to arrive at country, age and sex-specific multiplication factors that capture disability weights, the underlying case-mix (e.g. severe vs. mild disability, where applicable), and correction of burden estimates for co-morbidity, as used in the GBD 2010 study [2]. For NTDs with multiple sequelae (e.g. onchocerciasis) we followed the same procedure, but using a weight for each sequela based on an estimate of the average disability weight using GBD documentation (Table 1), because the YLD data provided by the GBD study did not separate the contributions of different sequelae. We treated all multiplication factors as constants. Remaining cases after 2010 were multiplied by the factors in the 2010-matrix, and for 1990–2010 an interpolation of the multiplication factors in both matrices was used. For counterfactual cases we used the multiplication factors in the 1990-matrix, or both matrices when 2010 was used as counterfactual (i.e. similar to the approach for remaining cases).

### Mortality calculations: Years of life lost (YLL)

Regarding our mortality calculations, we first translated GBD YLLs in 1990 and 2010 to actual country, age, and sex cause-specific mortality rates, using the age and sex-specific residual life expectancies as applied in the GBD study [1]. For HAT, VL and ascariasis, where mortality is closely linked (in time) to infection prevalence, these rates were treated as prevalent cases (of reversible sequelae) as described above and back-calculated to YLLs. For Chagas’ disease and schistosomiasis, where mortality is closely linked to late sequelae, we followed a different procedure. Similar to the calculation of YLDs for NTDs with multiple sequelae, we related YLLs in 1990 and 2010 to prevalent cases of selected sequelae, using a weight representing their lethality. For schistosomiasis, mortality was related to hematemesis (weight = 50), ascites (1.0) and schistosomiasis infestation (0.01). For Chagas’ disease, these were heart failure (10) and chronic heart disease (1.0).

### Special cases

Using the above method, some irreversible sequelae—in particular for Chagas’ disease and LF—showed for some countries values of *λ*(*a*,*t*) < 0, due to unrealistic fast declines in the GBD prevalence estimates between 1990 and 2010. Here, we chose alternative prevalences, but still within the confidence limit (Cl) provided by the GBD study, as follows. We reduced the GBD 1990 ‘Mean’ prevalence to 0.25 ‘Mean’ + 0.75 ‘Lower Cl’, and we increased the GBD 2010 ‘Mean’ prevalence to 0.25 ‘Mean’ + 0.75 ‘Upper Cl’.

The GBD 2010 estimates for leprosy appeared to be mistakenly based on overall leprosy new case detection (incident cases) instead of prevalence of (irreversible) cases with leprosy grade 2 disability, on which the disability weights are based. We therefore performed a recalculation to arrive at grade 2 disability prevalences as follows. First, we took from the WHO-published global leprosy data for 2010 the proportion of newly detected cases with grade 2 disability, which was 6% [12]. Secondly, prevalence of leprosy cases with grade 2 disability in virtual birth cohorts was accrued at a rate determined by this incidence density, while assuming a steady-state until 1990 and a linear decreasing incidence to 2010. We further assumed that excess mortality due to leprosy is negligible (μ* = 0.0). These prevalence values constituted the ‘GBD data’ on which our calculations were based.

## Results

**Fig 1:**
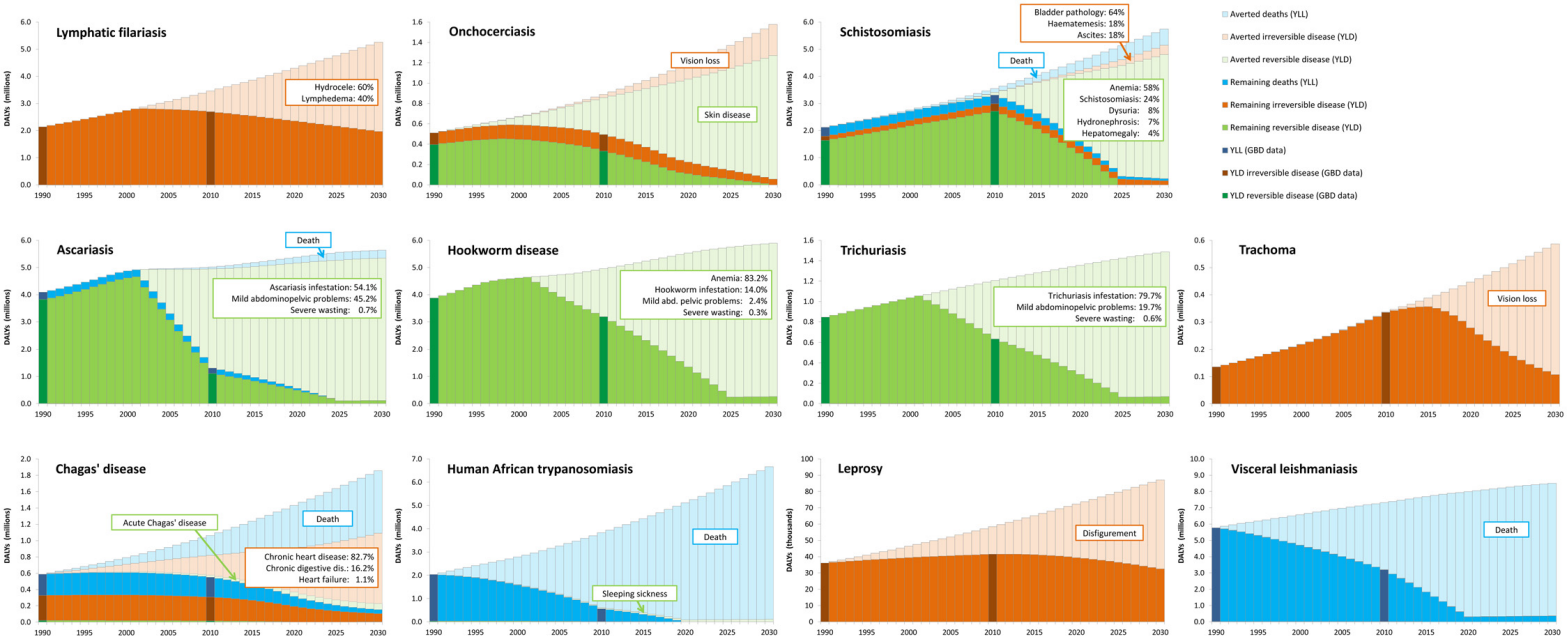
Global trends of remaining and averted disability adjusted life years (DALYs) from 1990 to 2030 for nine NTDs that are part of the London Declaration, with soil-transmitted helminths presented for ascariasis, hookworm disease and trichuriasis separately. DALYs are divided into years of life lost (YLL) and years lived with disability (YLD), with the latter subdivided into reversible and irreversible sequelae. Estimates for remaining DALYs were obtained by interpolating between 1990 (or the start-year of large-scale control efforts) and 2010, and further extrapolated until 2030, such that the 2020 targets of the WHO Roadmap were met. Averted DALYs were assessed by comparing the results of achieving the targets with the counterfactual, construed as the health burden had the 1990 (or 2010 if higher) situation continued unabated. The bars for 1990 and 2010 reflect the Global Burden of Disease (GBD) data [1–4], on which all calculations were based. The boxes indicate the most significant (i.e. visible) sequelae; where multiple sequelae make part of the total of reversible or irreversible disease (YLD) for an NTD, their relative contribution (%) to averted YLD in the period 2011–2030 is indicated. Underlying assumptions and more detailed results, both globally and at the country-level can be found here: https://erasmusmcmgz.shinyapps.io/dissemination/

Fig 1 shows the global trends in remaining and averted DALYs, distinguished into YLD of reversible and irreversible sequelae and YLL. According to the original GBD 2010 data (dark-colored bars), the health burden of onchocerciasis, STH, Chagas’ disease, HAT and VL has clearly decreased over the period 1990 to 2010. For LF, schistosomiasis and leprosy, the absolute burden has increased, but not as fast as would be expected from the counterfactual. Thus, for these NTDs, the relative burden has decreased, when correcting for population growth. Only for trachoma (and in some countries for schistosomiasis), the GBD-estimated burden has increased faster than would be expected from the demographic trends over the period 1990–2010.

Meeting the 2020 targets will lead to a substantial health-impact for all NTDs (Fig 1). It is clearly visible that reversible sequelae (green) are disappearing faster than irreversible sequelae (brown). This makes the health impact of reaching the targets for LF, trachoma and leprosy over the first two decades somewhat less spectacular compared to that for the other NTDs, of which the burden is mainly caused by reversible sequelae or death. Another important factor determining the overall health impact is population growth and other demographic developments, as expressed by the counterfactual. NTDs that are prevalent in Asia (LF, STH, leprosy and VL) show a slower rise of the counterfactual compared to the NTDs mainly confined to Africa (onchocerciasis, trachoma and HAT) or South America (Chagas’ disease).

**Fig 2:**
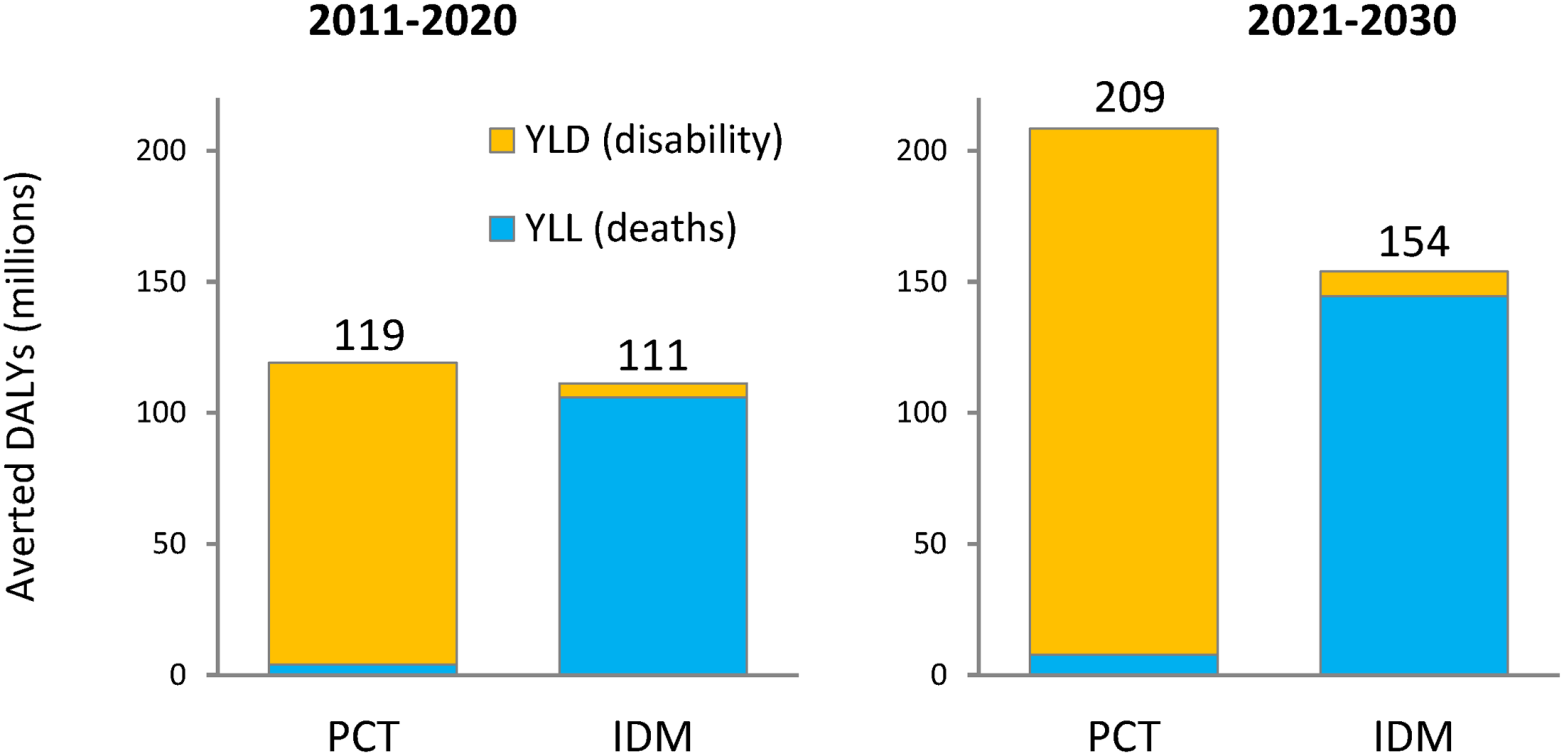
Estimated overall health impact of meeting the WHO Roadmap targets for nine London Declaration NTDs. Five (lymphatic filariasis, onchocerciasis, schistosomiasis, soil-transmitted helminths and trachoma) are to be controlled by preventive chemotherapy (PCT), and four (Chagas’ disease, human African trypanosomiasis, leprosy and visceral leishmaniasis) by innovative and intensified disease management (IDM). Values are in millions of averted DALYs per 10-year period, subdivided into years of life lost (YLL) and years lived with disability (YLD).

Overall, meeting the targets of London Declaration NTDs will avert about 600 million DALYs in the two decades after 2010, nearly equally distributed between PCT and IDM-NTDs, with the former mostly (96%) attributable to averted disability, whereas the latter largely (95%) results from averted premature death (Fig 2). These health gains include about 150 million averted irreversible disease manifestations, in particular chronic heart disease due to Chagas’ disease, bladder pathology due to schistosomiasis, and hydrocele and lymphedema due to LF (Table 3). In addition, approximately 5 million deaths are averted, mainly from VL and HAT, and to a lesser extent Chagas’ disease (Table 4).

**Table 3:** Estimated number of averted new cases of irreversible disease (in millions) when meeting the WHO Roadmap targets for London Declaration NTDs.

NTD	Sequela	2011–2020	2021–2030
Lymphatic filariasis	Lymphedema	8.4	10.3
	Hydrocele due to lymphatic filariasis	12.7	15.0
Onchocerciasis	Vision loss due to onchocerciasis	1.8	3.3
Schistosomiasis	Hematemesis due to schistosomiasis	0.1	0.4
	Ascites due to schistosomiasis	0.3	0.9
	Bladder pathology due to schistosomiasis	10.9	31.9
Trachoma	Trachoma	3.2	6.8
Chagas’ disease	Chronic heart disease due to Chagas’ disease	14.6	22.7
	Chronic digestive disease due to Chagas’ disease	1.3	1.7
	Heart failure due to Chagas’ disease	0.1	0.2
Leprosy	Disfigurement due to leprosy	1.0	1.3
Total averted new cases		54.3	94.6

**Table 4:** Estimated number of averted deaths (in millions) when meeting the WHO Roadmap targets for the five London Declaration NTDs with associated mortality.

NTD	2011–2020		2021–2030	
Visceral leishmaniasis	0.99	50.7%	1.36	47.6%
HAT	0.70	35.7%	0.99	34.6%
Chagas’ disease	0.16	8.3%	0.29	10.2%
Schistosomiasis	0.08	4.2%	0.18	6.3%
STH—Ascariasis	0.02	1.1%	0.04	1.3%
Total averted deaths	1.96	100.0%	2.87	100.0%

## Discussion

Of the 600 million DALYs overall averted in the period 2011–2030, in the ideal situation of meeting the WHO Roadmap targets of London Declaration NTDs, about 30 million will be realized in the year 2020, increasing to 40 million in the year 2030. This is of the same order of magnitude as the current annual health burden of any of the ‘big three’ infectious diseases, HIV/AIDS, TB and malaria, which accounted for about 80, 50 and 80 million DALYs, respectively, in 2010 [3]. Clearly, for these three infections elimination is a more remote perspective than for the nine NTDs targeted by the London Declaration. Thus, the ongoing efforts to control the big three seem to justify similar investments in NTD control. In addition, it can be expected that for several of these NTDs control efforts will lead to a cessation of transmission over vast regions, after which further control can be discontinued and investments wound down adding to the value of this investment for future generations.

STH accounts for one-third (34%) of the averted DALYs, almost entirely due to avoided disability. This perhaps surprising finding can be easily explained by the wide-spread distribution of STH [13]. Importantly, approximately half (46%) of the averted STH-burden would be realized in China. This brings to the fore the sensitivity of our results to the choice of counterfactual. That is, our assumption that the situation of 1990 would continue unabated may be questioned for several countries, including China, which have experienced unprecedented economic and social development over the past decades [14]. For example, the health impact for STH would be about halved if the situation in 2010 were used as the counterfactual, as can roughly be concluded from Fig 1, but such a drastic correction would certainly not be reasonable for many endemic countries in Africa and Southern Asia. On the other hand, socioeconomic development may also have facilitated the spread of NTDs, in particular schistosomiasis, of which large outbreaks followed the construction of dams and irrigation schemes [15]. HAT perhaps follows more erratic patterns, reflecting e.g. civil unrest, war and also ecological circumstances [16], so that the year 1990 may not be representative of the actual counterfactual over 1990–2020. Trachoma and schistosomiasis showed large increases in GBD prevalence from 1990 to 2010, which may well reflect an underestimation of the 1990 burden. Consequently, this may have led to underestimating both the counterfactual and the health impact. Another potential source of underestimation of the health impact for some NTDs may be that the largest gains are achieved in the initial years of programs, followed by a slow down towards the target year, as it becomes harder to reach the more marginalized populations. Furthermore, by using a fixed excess mortality rate μ* for irreversible sequelae (where applicable) we may have somewhat overestimated health impacts for these sequelae as treatment is likely to improve over time. However, since the remaining cases get older at the same time, possibly experiencing a higher mortality, we may have introduced some underestimation as well. Clearly, by using a uniform methodology we have introduced (perhaps occasionally substantial) under or overestimation of NTD and country-specific results, but we are confident that the overall bias in our estimated health impact of reaching the targets will be small.

Almost half (44%) of the overall health impact is attributable to averted deaths, in particular from visceral leishmaniasis and HAT, and to a lesser extent Chagas’ disease, followed by schistosomiasis and STH (ascariasis). In our calculations, we followed the GBD accounting philosophy which assigns all DALYs (i.e. residual life expectancy at the age of death) resulting from a death to the year in which it occurred [1], whereas DALYs attributable to morbidity are accrued during the years that individuals suffer [2]. Moreover, remaining life expectancies were based on the demography of Japan, according to the fundamental concept that all people are entitled to the best life expectancy in the world, irrespective of e.g. country of residence and socioeconomic status. Clearly, other methodologies might have distributed health gains differently over time.

Our calculations depend strongly on the estimates made in the GBD study [1–3]. These estimates are notably uncertain for NTDs, given the paucity of data on their geographic spread and control. Most GBD 1990 and 2010 estimates for NTDs show very wide confidence intervals, often ± 50% the mean, but sometimes with an upper confidence limit up to 5 times the mean. As a consequence, our predictions (all based on GBD point estimates) are subject to at least a similar degree of uncertainty. Also, the GBD disability weights used are still under heavy debate, such as the relatively low value for blindness as compared to itching [17]. Furthermore, our calculations are confined to the 31 sequelae considered in the GBD study, and discussions continue about whether additional sequelae need to be considered. In particular, the choice not to include so-called subtle morbidities, such as impaired cognitive development due to STH and schistosomiasis, or poor mental health from stigma and discrimination due to the disfigurements caused by LF and leprosy, is considered an important omission by many [13,18–21]. Our results also depend upon the interpretation and formulation of the WHO Roadmap targets [7,9], which occasionally are ambiguous. Consulting disease experts at WHO has resulted in agreement about interpretations for most NTDs, even though sometimes the targets were considered too general or utopic.

In addition to the intrinsic value of averting human suffering and death, this health impact of reaching the targets will also give rise to major economic and societal improvements, such as increased productivity and avoided (often catastrophic) out of pocket payments for treatment and care, which can be assigned monetary values. In particular, the currently ignored subtle morbidities are likely responsible for major societal impacts.

We realize that the targets are ambitious, and may for instance be jeopardized by challenges in drug distribution, disease surveillance and health care access. Also, systematic non-compliance in mass-drug administration, population groups currently not eligible for treatment, and development of drug or insecticide resistance could be serious threats, as demonstrated in a recent collection of studies by the NTD Modelling Consortium focusing on the question whether we are on track to reaching the goals [22]. Furthermore, even if the targets are reached by 2020 it is essential that control and surveillance are continued to avoid rebounding effects, certainly for those NTDs where elimination of transmission cannot be expected.

In conclusion, NTDs together constitute a major health burden, comparable to any of the three major infectious diseases HIV/AIDS, TB, and malaria. Achieving internationally agreed targets of NTD control and elimination will bring about major gains in health and reductions in human suffering. Much of this will be achieved by avoiding morbidity rather than mortality as many of the parasites involved, such as soil transmitted helminths, rarely kill their hosts. This also implies that our impact assessment depends on the valuation of health states as used by GBD, a valuation that inevitably is somewhat subjective and open to debate. We did not consider the costs involved in reaching these targets, but a recent assessment demonstrated that these are relatively modest [23], indicating that the cost-effectiveness of interventions to control NTDs will likely be high. One thing is certain however: as NTDs are disorders that disproportionately affect the poor, their control will considerably improve global equity.

## References

[1] Lozano R, Naghavi M, Foreman K, Lim S, Shibuya K, et al (2012) Global and regional mortality from 235 causes of death for 20 age groups in 1990 and 2010: a systematic analysis for the Global Burden of Disease Study 2010. Lancet 380: 2095–2128. 10.1016/S0140-6736(12)61728-0 23245604 PMC10790329

[2] Vos T, Flaxman AD, Naghavi M, Lozano R, Michaud C, et al (2012) Years lived with disability (YLDs) for 1160 sequelae of 289 diseases and injuries 1990–2010: a systematic analysis for the Global Burden of Disease Study 2010. Lancet 380: 2163–2196. 10.1016/S0140-6736(12)61729-2 23245607 PMC6350784

[3] Murray CJL, Vos T, Lozano R, Naghavi M, Flaxman AD, et al (2012) Disability-adjusted life years (DALYs) for 291 diseases and injuries in 21 regions, 1990–2010: a systematic analysis for the Global Burden of Disease Study 2010. Lancet 380: 2197–2223. 10.1016/S0140-6736(12)61689-4 23245608

[4] Hotez PJ, Alvarado M, Basáñez M-G, Bolliger I, Bourne R, et al (2014) The Global Burden of Disease Study 2010: interpretation and implications for the neglected tropical diseases. PLoS Negl Trop Dis 8: e2865 10.1371/journal.pntd.0002865 25058013 PMC4109880

[5] Hotez PJ, Fenwick A, Savioli L, Molyneux DH (2009) Rescuing the bottom billion through control of neglected tropical diseases. Lancet 373: 1570–1575. 10.1016/S0140-6736(09)60233-6 19410718

[6] Conteh L, Engels T, Molyneux DH (2010) Socioeconomic aspects of neglected tropical diseases. Lancet 375: 239–247. 10.1016/S0140-6736(09)61422-7 20109925

[7] World Health Organization (2012) Accelerating work to overcome the global impact of neglected tropical diseases: a roadmap for implementation (http://www.who.int/neglected_diseases/NTD_RoadMap_2012_Fullversion.pdf; accessed 16 March 2015).

[8] London Declaration on Neglected Tropical Diseases (http://unitingtocombatntds.org/sites/default/files/resource_file/london_declaration_on_ntds.pdf; accessed 16 March 2015).

[9] World Health Organization (2013) Sustaining the drive to overcome the global impact of neglected tropical diseases: second WHO report on neglected tropical diseases (http://www.who.int/neglected_diseases/9789241564540/en/; accessed 16 March 2015).

[10] Institute for Health Metrics and Evaluation (http://www.healthdata.org/gbd; accessed 16 March 2015).

[11] United Nations Department of Economic and Social affairs, Population Division, Population Estimates and Projections Section (http://esa.un.org/unpd/wpp/unpp/panel_population.htm; assessed 10 April 2015).

[12] World Health Organization (2011) Leprosy update, 2011. Wkly Epidemiol Rec 86, 389–399. 21887885

[13] Pullan RL, Smith JL, Jasrasaria R, Brooker S (2014) Global numbers of infection and disease burden of soil transmitted helminth infections in 2010. Parasit Vectors 7: 37 10.1186/1756-3305-7-37 24447578 PMC3905661

[14] The World Bank, China, Overview (http://www.worldbank.org/en/country/china/overview; accessed 12 November 2015).

[15] N'Goran EK, Diabate S, Utzinger J, Sellin B (1997) Changes in human schistosomiasis levels after the construction of two large hydroelectric dams in central Côte d'Ivoire. Bull World Health Organ 75: 541–545. 9509626 PMC2487023

[16] Mhlanga JD (1996) Sleeping sickness: perspectives in African trypanosomiasis. Sci Prog 79: 183–214. 8973165

[17] Coffeng LE, Stolk WA, Zouré HGM, Veerman JL, Agblewonu KB, et al (2014) African Programme for Onchocerciasis Control 1995–2015: updated health impact estimates based on new disability weights. PLoS Negl Trop Dis 8: e2759 10.1371/journal.pntd.0002759 24901642 PMC4046979

[18] Miguel E, Kremer M (2004) Worms: identifying impacts on education and health in the presence of treatment externalities. Econometrica 72: 159–217.

[19] Baird S, Hicks JH, Kremer M, Miguel E (2012) Worms at work: long-run impacts of child health gains. Harvard University Working Paper (http://scholar.harvard.edu/files/kremer/files/klps-labor_2012-03-23_clean.pdf; accessed 16 March 2015).

[20] King CH, Dickman K, Tisch DJ (2005) Reassessment of the cost of chronic helminthic infection: a meta-analysis of disability-related outcomes in endemic schistosomiasis. Lancet 365: 1561–1569. 15866310 10.1016/S0140-6736(05)66457-4

[21] Litt E, Baker MC, Molyneux DH (2012) Neglected tropical diseases and mental health: a perspective on comorbidity. Trends Parasitol 28: 195–201. 10.1016/j.pt.2012.03.001 22475459

[22] Hollingsworth D (ed.) (2015) Quantitative analysis of strategies to achieve the 2020 goals for neglected tropical diseases: where are we now? Parasit Vectors 8 (article collection) (http://www.parasitesandvectors.com/series/ntdmodels2015; accessed 2 December 2015).

[23] Seddoh A, Onyeze A, Gyapong JO, Holt J, Bundy D (2013) Towards an investment case for neglected tropical diseases: including new analysis of the cost of intervening against preventable NTDs in sub-Saharan Africa. The Lancet, CIH Working Paper, July 2013 (http://globalhealth2035.org/sites/default/files/working-papers/towards-an-investment-case.pdf; accessed 16 March 2015).

[24] Salomon JA, Vos T, Hogan DR, Gagnon M, Naghavi M, et al (2012) Common values in assessing health outcomes from disease and injury: disability weights measurement study for the Global Burden of Disease Study 2010. Lancet 380: 2129–2143. 10.1016/S0140-6736(12)61680-8 23245605 PMC10782811

[25] World Health Organization, PCT databank (http://www.who.int/neglected_diseases/preventive_chemotherapy/lf/en/; accessed 7 April 2015).

[26] African Programme for Onchocerciasis Control (WHO/APOC) (2013). Revised plan of action and budget 2014–2015: Elimination of onchocerciasis in Africa. JAF document 19.9 (November 2013)(http://www.who.int/apoc/about/structure/jaf/Final_Communique_JAF19_Final_English_140114.pdf?ua=1; accessed 7 April 2015).

[27] World Health Assembly (2001) WHA 54.19, Schistosomiasis and soil-transmitted helminth infections (http://www.who.int/neglected_diseases/mediacentre/WHA_54.19_Eng.pdf; accessed 7 April 2015).

[28] World Health Organization (2013) Global Alliance for the Elimination of Blinding Trachoma by 2020: Progress report on elimination of trachoma, 2012. Wkly Epidemiol Rec 88, 242–251 (http://www.who.int/wer/2013/wer8824.pdf?ua=1; accessed 7 April 2015).

[29] World Health Organization (2013) Report of the 17th meeting of the WHO alliance for the global elimination of blinding trachoma. Geneva, 22–24 April 2013 (http://www.who.int/blindness/publications/GET17Report_final.pdf?ua=1; accessed 10 April 2015).

[30] Tarleton RL, Gürtler RE, Urbina JA, Ramsey J, Viotti R (2014) Chagas disease and the London Declaration on neglected tropical diseases. PLoS Negl Trop Dis 8: e3219 10.1371/journal.pntd.0003219 25299701 PMC4191937

[31] World Health Organization, Human African trypanosomiasis: The current situation (http://www.who.int/trypanosomiasis_african/country/country_situation/en/; accessed 7 April 2015).

[32] Moore EM, Lockwood DN (2010) Treatment of visceral leishmaniasis. J Global Infect Dis 2: 151–158. 10.4103/0974-777X.62883 PMC2889655 20606971

